# Shu-szi Sin: A Chinese pioneer biologist, ancient agronomist, and educator

**DOI:** 10.1007/s13238-019-00657-x

**Published:** 2019-09-11

**Authors:** Qun Xie, Yan-ping Cheng, Lei Fu

**Affiliations:** Teacher Education College, Zhejiang Normal University, Jinhua, 321000 China

Shu-szi Sin (辛树帜, 1894–1977) (Fig. 1), a Chinese pioneer biologist and ancient agronomist, initiated the earliest scientific biological investigation of the Dayao mountain in both Guangxi and Guangdong provinces, discovering more than 20 new species that have been acknowledged internationally. He was one of the founders and initiators of the China Botanical Society and the China Zoological Society. Mr. Sin was also a great educator. Among one of those who first introduced the scientific method into agricultural history research, Mr. Sin helped to set up the National Northwest Agriculture and Forestry College (now Northwest A&F University) and the Lanzhou University. He devoted himself to the development of higher education in Northwest China.

**Figure 1:**
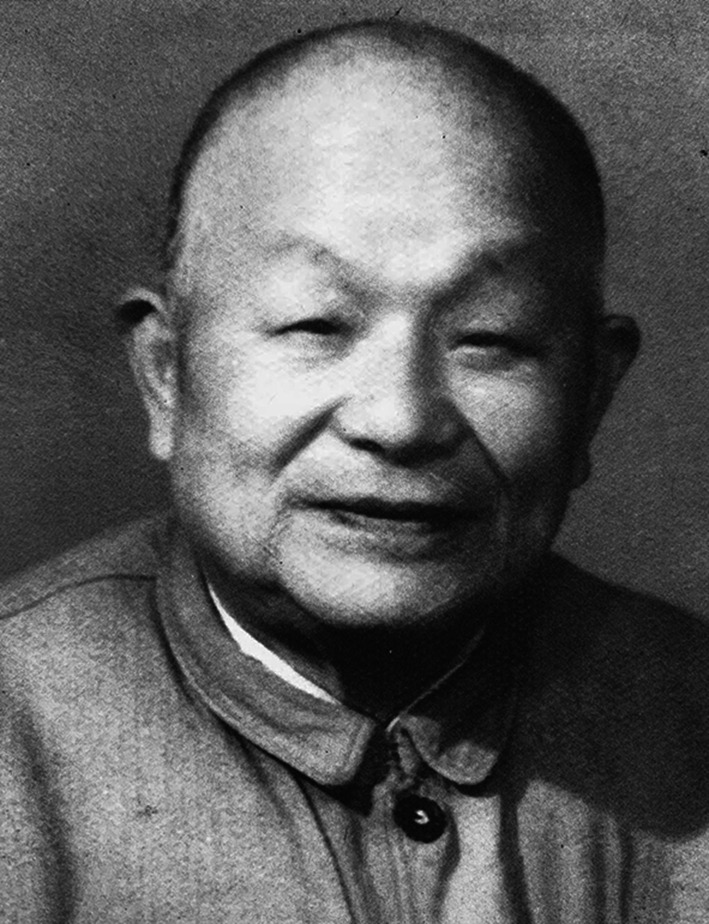
Shu-szi Sin (1894–1977)

Shu-szi Sin was born in a poor peasant family in Linli County, Hunan Province in 1894. At the age of 9, he went to study in a traditional Chinese private school.^1^ In 1910, he entered the Hunan West Road Normal Academy (now Changde No. 1 Middle School). In the autumn of 1915, he was admitted to the National Wuchang Higher Normal School (now Wuhan University) and began to study biology. After graduation, he taught at the Changsha Mingde Middle School and the Hunan Provincial First Normal School. From 1924 to 1927, he went to Europe to study at his own expenses. At first, he studied at the London University. Then, he went to the University of Berlin and became a student of Dr. Diels, an expert in plant taxonomy who was also familiar with plant taxonomy in China. Dr. Diels told Sin that he could contribute to the classification of animals and plants in Guangdong and Guanxi, as no research studies had been conducted since then on this field.

In 1927, Sin Shu-szi returned to China. In Fu Ssu-nien’s (傅斯年) referral, Mr. Sin became a professor and Director of the Department of Biology of the Sun Yat-Sen University. Influenced by Dr. Diels, Sin Shu-szi presided over the scientific investigation of the Dayao mountain in Guangxi together with his colleagues in May 1928, where they collected more than 30,000 specimens. In november of the same year, he and his colleagues conducted a larger field study at the Dayao mountain, which lasted until February 1929. Soon after, Mr. Sin led a third scientific investigation of the Dayao mountain, opening a precedent for large-scale scientific investigation and biological collection in China. The scope of its investigation and collection far exceeded the Yaoshan area, involving the Yunwu, Doupeng, and Fanjing mountains in Guizhou; the Jintong mountain in southern Hunan; Beijiang, Yongchang, and Yaoshan in the Guangdong Province; and the Hainan province.

Mr. Sin and his colleagues collected more than 60,000 specimens, including 30,000 specimens of plants, over 100 specimens of 40 species of mammals, about 4,000 specimens of 210 species of birds, around 500 specimens of 40 species of reptiles, and more than 2,000 specimens of birds. Apart from revealing the treasures of flora and fauna in southern China, he also identified nearly 20 new species, such as the *Shinisaurus crocodilurus*, the *Sinia rhodoleuca*, and the *Corybas sinii* etc. Based on their investigations and collections, a relatively complete animal and plant specimen room was established in the Sun Yat-sen University, which became an important basis and resource to cultivate and attract a large number of specialized personnel engaged in animal and plant research, enhancing the academic influence of China in the international biology community.

In the Dayao mountain study, Mr. Sin and his colleagues broke the barriers between science and humanities, by using breakthrough scientific research methods in the humanities to investigate and report the life and customs of the Yao minority. Based on field visits and interviews with villagers, a map of the village was drawn. In addition, Mr. Sin also produced a considerable quantity of notes on local customs and habits, and compiled a large number of ethnic folklore materials such as the *Yaoshan Two-Month Inspection Record*, the *Zheng Yao Dance Song*, the *Ki Zi Ge*, and the *Yaoshan Collection Schedule*. Thanks to their efforts, the people and the life of the Yao minority were introduced to, and accepted by, the outside world.

In 1932, Mr. Sin served as the head of the editorial department of the Ministry of Education of the National Government. The next year, the editorial office was expanded as the National Translator-Editor Center, of which Mr. Sin was the first director. He believed that standardizing the scientific terminology was an important foundation for the development of natural sciences in China. Accordingly, the National Translator-Editor Center began to translate and edit the terminology in chemistry, astronomy, geology, medicine, physics, and other fields. In order to facilitate this work, Mr. Sin and his colleagues also enrolled a number of experts and organized a series of academic conferences. The standardization of the scientific terminology in the first years of 1930 gave a crucial contribution to the development of science in China.

Realizing the importance of building a scientific community, Mr. Sin built good relationships with other scholars. He was one of the founders and sponsors of the Botanical Society of China, whose preparation and initiation began in mid-summer in 1933. As one of the initiators, Mr. Sin attended the inaugural meeting in Chongqing, when the Constitution of the Chinese Botanical Society was adopted, and Mr. Sin was recommended as a key member (Sun, 2003). The establishment of the Chinese Botanical Society marks the beginning of a new stage in the development of modern botany in China. In 1934, Mr. Sin, Mr. Zhi Bing (秉志), Mr. Deyu Xue (薛德育) and others initiated the establishment of the China Zoological Society, of which Mr. Sin was nominated as Vice Chairman. The establishment of both the Botanical Society and the Zoological Society provided important platforms for communication among biologists.

In 1932, Mr. Sin visited Luoyang and other cities of northwest China, where he found the absence of higher agricultural schools. Therefore, he felt that he should do something to improve the situation in that region. Mr. Sin believed that Wugong, a city in the Shaanxi province, was an important birthplace of ancient Chinese agriculture; for this reason, he advocated the establishment of agricultural colleges in that city. At the end of 1932, he participated in the founding of the Northwest Agriculture and Forestry College in Wugong. In July 1936, the preparation work for the Northwest Agriculture and Forestry College was over. In spite of the harsh conditions in the northwest, he resigned from the position of Director of the National Translator-Editor Center, serving as President of the Northwest Agriculture and Forestry College and, later, as Dean of the Northwest Agricultural College. During that time, he regularly held lectures with teachers and students every week, listening to opinions, and insisting on doing exercises with students in the morning. Therefore, he was not only familiar with the teaching situation, but also knew well teachers and even most students. Many of his students later became the backbone of the agricultural field. The teachings of the old Dean were appreciated.

In 1940, Mr. Sin left Shaanxi and became professor and Director of the Central University in Chongqing. He was also elected as member of the National Political Participation. Due to the illness of his mother, he resigned his various duties and returned home village to take care of his mother. During his stay in the countryside, he was deeply impressed by its economic and cultural backwardness. He persuaded the government to support education, and participated in the preparation of the Hunan 14th Middle School, the Xuwu Middle School, and the Jiuli Middle School in the Hunan Province. In 1945, Mr. Sin was elected as President of the Hunan Provincial Education Association.

On March 26, 1946, Shu-szi Sin was appointed by the government of the Republic of China as principal, responsible for the preparatory work of the Lanzhou University. He believed that running a comprehensive university, composed of five colleges, in Lanzhou was crucial to improve the education of the whole northwest. To make Lanzhou University the capital of northwest education, he first focused on the establishment of the faculty, emphasizing the need to run the veterinary college, the Tibetan language department, and the Russian department, among others, and to train qualified graduates that were urgently needed for the development of the northwest economy and culture. Secondly, he managed to attract scholars at the Lanzhou University. Due to the poor transportation and living conditions of northwest China at that time, few scholars were willing to move to Lanzhou. Mr. Sin visited some scholars in Beijing, Shanghai, and Guangdong, and invited them to the Lanzhou University as visiting professors or short-term lecturers. Thanks to his good reputation and good relationships with other scholars, the Lanzhou University attracted a number of scholars around China. His third contribution was the purchase of a large quantity of books and teaching equipment. With his recommendation, a large number of Chinese and foreign books and academic journals (totally more than 100,000 volumes) were collected in the Lanzhou University Library, which in just three years jumped at the top of the northwest colleges and universities.

After the founding of the People’s Republic of China, Mr. Sin returned as Dean of the Northwest Agricultural College. He deeply felt the importance of investigating the agronomic heritage of China. In 1952, he established the ancient agronomy research team, which included scholars such as Shenghan Shi (石声汉), Weiying Xia (夏纬瑛), Yao Zhou (周尧), and Yunti Cui (崔允褆). Although there was no funding support at that time, all team members were working hard. In 1965, their efforts were recognized by the government, and the Ancient Agricultural Research Laboratory was established at the Northwest Agricultural College. They studied and sorted the ancient agricultural literature, and addressed the issue of soil and water conservation. They gained experience in studying the ancient agricultural literature, especially in aspects such as the identification of authenticity, the examination of the origins of the literature, and the punctuation, subsection, annotation, and translation (Zhang and Pu, 2012). Under Mr. Sin’s auspices, the laboratory overcame many difficulties, examining dozens of ancient books, publishing monographs of millions of words, and was highly valued and praised by scholars at home and abroad (Fig. 2).

**Figure 2:**
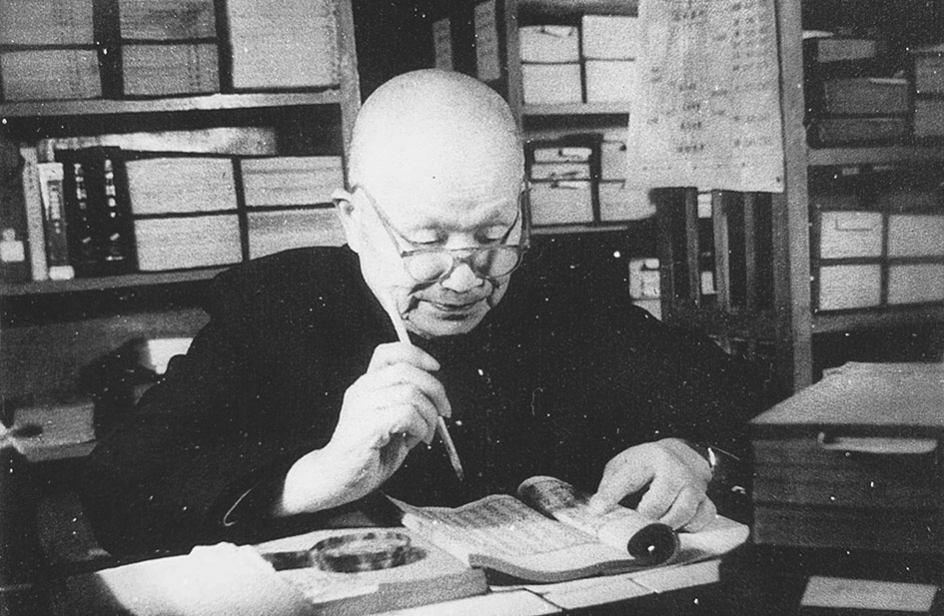
Mr. Shu-szi Sin working in the Ancient Agricultural Research Laboratory in 1973

Mr. Sin’s research outcomes include: The *Study of Chinese Fruit Trees History*; the *New Analysis of Yu Gong*; the *Study on the* *Book* *of* *Change*; and the *Preliminary discussion on 159 cultivated plants in the book of agricultural administration.* The *Study of Chinese Fruit Trees History* systematically analyzes and compares the achievements of the earliest fruit trees cultivation in China, according to the records of fruit trees from the Western Zhou Dynasty to the end of the Tang Dynasty, and carefully examines the types and names of fruit trees during this period. In the *New Analysis of Yu Gong*, Mr. Sin conducts a scientific investigation of “water and soil” and “tribute” such as comb and bag which recorded in *Shang Shu · Yu Gong*, an ancient article describing the geography, establishing an example of ancient agronomy research. In his later years, Mr. Sin paid great attention to the issue of soil and water conservation in China, especially in the northwest region. He published the *Historical Research on Soil and Water Conservation in China*, and presided over the preparation of the *Introduction to Soil and Water Conservation*. In order to collect first-hand information on vegetation damage and soil erosion, he conducted field studies in Shaanxi, Yunnan, and other places. Having reached the age of eighty, he was overworked. When he returned from the Yunnan investigation, he fell ill. He passed away on October 24, 1977.

As a biologist, ancient agronomist, and educator, Mr. Shu-szi Sin was always passionate in his work and research. His work was visionary and groundbreaking. He loved his motherland, and devoted all himself to the development of science and education, especially in northwest China.

## Footnotes

1 Traditional Chinese Private School (私塾) is a kind of small scale school which was run and taught by educated persons. The schools are set by the teachers in their own homes. The students are mostly children aged six to eight nearby.
